# Practice towards care and maintenance of peripheral intravenous cannula among nurses and midwives in teaching hospitals, Amhara, Ethiopia

**DOI:** 10.11604/pamj.2024.48.166.43279

**Published:** 2024-08-09

**Authors:** Demewoz Kefale, Shegaw Zeleke Baih, Yeshiambaw Eshetie Ayenew

**Affiliations:** 1 Department of Pediatrics and Child Health Nursing, College of Health Sciences, Debre Tabor University, Debre Tabor, Ethiopia,; 2 Department of Adult Health Nursing, College of Health Science, Debre Tabor University, Debre Tabor, Ethiopia

**Keywords:** Peripheral intravenous cannulation, practice, nurses, midwives, hospitals

## Abstract

**Introduction:**

nearly 80% of hospital patients called for a peripheral intravenous catheter (PIVC) for important medications and fluid therapies. Peripherally inserted intravenous cannulation is the most common procedure done in patient care delivery. Even though insertion of IV cannula is essential and common, there are different limitations starting from insertion without vibrant indication to improper management and care of these devices. Patients can experience multiple adverse events during the insertion. The objective of this study was to investigate the care of peripheral intravenous cannulation by nurses and midwives in the Amhara Region, Ethiopia, 2022.

**Methods:**

an institutional-based cross-sectional study was conducted. Data was collected using structured interviewer-administered questionnaires plus an observational checklist. A simple random sampling technique was used to select 415 nurses and midwives. Data were entered using Epi Data version 3.1 and analyzed using SPSS version 25. Bi-variable and multivariable analyses were conducted to examine the association between independent and outcome variables.

**Results:**

the findings of this study revealed that 4.9% of respondents had good practice for the care of peripheral intravenous cannulation. Work experience (AOR= 7.269, 95% CI= 1.68, 31.40), having special training on care of peripheral intravenous cannulation (AOR= 10.12, 95% CI= 4.32, 19.45) and presence of protocol guideline (AOR=3.31, 95% CI= 2.83, 12.87) were significantly associated with good practice on the care and maintenance of peripheral intravenous cannulation.

**Conclusion:**

findings showed that poor peripheral intravenous cannulation practice and care which was predicted by work experience, existence of guidelines, and training. As a result, Nurses and midwifes must keep up to speed with focused in-service training, access, and/or follow thorough protocol guidelines for the management of peripheral intravenous cannulation.

## Introduction

Nearly 80% of hospital patients called for a peripheral intravenous catheter (PIVC) for important medications and fluid therapies [1,2]. Intravenous cannulation is a technique or procedure in which a cannula is placed inside a vein to provide different medication with the insertion of a peripheral Cannula into the venous system [3]. A peripheral intravenous cannula is defined as a device made of biocompatible material that connects the patient´s bloodstream to the external environment, its terminal part is located in any tributary vessel of the upper or lower vena cava [4,5].

When deciding on the best PIVC for the patient, factors to be considered include the prescribed therapy or treatment regimen; anticipated duration of therapy; vascular characteristics and patient´s age, co-morbidities, history of infusion therapy, and ability and resources available to care for the device [6]. It is important to recognize that most patients in hospitals must receive at least one peripheral intravenous (IV) cannula because they are candidates for parenteral medications, fluid resuscitation, or blood transfusion. So for better outcomes and to reduce the risk of complications health professionals especially nurses and midwives should realize the right size cannula selection, proper placement, and care for these devices [7].

Nurses and midwives can be identified in the lack of specialized personnel, an insufficient level of training, inadequate knowledge of devices, particularly the most recent ones, and inadequate application of assessment procedures [4]. The studies indicated peripheral IV cannula fail 20%-69% of the time with complications that lead to pain, patient dissatisfaction, depletion of venous access, and increased costs in treating minor and major complications [3,8]. The simple procedure of PIV cannulation can lead to catastrophic complications like septic pulmonary emboli and widespread cellulitis if not done with proper care and precautions [9]. Short peripheral cannula thrombophlebitis (SPCT) occurs in most hospitalized patients, symptoms appear on average 3 days after cannula insertion and can lead to extended hospitalization and increased Competence in peripheral vein cannulation (PVC) in nursing and midwifery is emphasized as important to ensure effective treatment and patient safety. Studies show the presence of a practice gap among hospital nurses and midwives towards care and maintenance of PIVC and related complications [6,10]. Generally, these professionals are not practicing aseptic technique during insertion, have poor documentation (insertion date, site, frequency of cannula change and cannula size) they did not practice daily care after insertion; they do not commonly flush with normal saline to maintain patency of the cannula [11]. Their practice of providing medications and fluids by reusing the infusion set after once detached from the cannula is common, which is very important for easy access of microorganisms to the bloodstream [12].

Our experience revealed that nurses and midwives in our setting are not practicing according to protocol nurses and midwives have failed to give care (attention) for none touch areas like the tips of the intravenous set, a tip of the cannula. In the absence of a sterile field, patients complaining of pain at the cannula site commonly, there can observe untidy cannula sites from patients, which indicates inadequate patient education about the care of the cannula site, lack of periodic assessment, and poor practice of nurses and midwives. The objective of this study was to investigate the care of peripheral intravenous cannulation by nurses and midwives in the Amhara Region, Ethiopia.

## Methods

**Study setting and design:** Debre Tabor and Felegehiwot Referral Hospitals are found in Amhara Regional State North West Ethiopia. Debre Tabor hospital is found in South Gondar Zone and serves more than 2 million people and Felegehiwot hospital is found in Bahir Dar which serves for West Gojjam and Awi Zone for Bahir Dar city administrative w *oreda*. There were 671 nurses and midwives in Debre Tabor Referral Hospitals (DTRH) and Felegehiwot Referral Hospital (FHRH) during the study period. The study was conducted between March 15^th^ and April 30^th^, 2022. Institutional-based cross-sectional study design was used.

**Population and sample size:** all (N=671) nurses and midwives who were working in DTRH and FHRH hospitals were the study population. The sample size was calculated as P= level of practice towards care and maintenance of PIVC in the previous study=43.3% [13], 95% confidence level (Zα/2=1.96) and absolute precision or margin of error was 5% (d=0.05). By adding a 10% non-response rate the total sample size was 415.

**Sampling procedure:** the total number of nurses and midwives from each hospital has been determined. The calculated sample size in each hospital was proportionally allocated to the number of nurses and midwives and the study unit was selected using a simple random sampling technique from the work schedule checklist of professionals. So, based on the proportional allocation about 229 nurses and 27 midwives from FHRH and 136 nurses and 23 midwives from DTRH were selected. Consequently, 256 nurses and midwives from FHRH, and 159 nurses and midwives from DTRH were selected, adhering to the proportional allocation method.

**Study variables, tools, and data collection procedure:** the outcome variable was the practical care of peripheral intravenous cannulation. The data were collected using structured self-administered questionnaires plus an observational checklist. The questionnaire was prepared in English language and included respondents´ socio-demographic characteristics and practice related questions, and checklists. The tools were validated and adapted from the previous study with in-depth literature review [10,14-16]. The questionnaire was checked thoroughly for objectivity and variable assessment before it was distributed to the data collectors. Half-day training was given to the data collectors and the supervisors on the study protocol, including the study objectives, the relevance of the study and confidentiality of information, respondents´ rights, and informed consent. One supervisor was a nurse holding a Master's Science degree and two data collectors were nurses holding a Bachelor of Science degree. Participants who scored above and equal to 70% on practice questions were considered to have good practice, whereas participants who scored below 70% were considered to have poor practice for the care of intravenous cannulation [13]. In this study, appropriate veins mean based on American CDC recommendations; insertion site for short and midline peripheral catheters as the upper extremity veins for adults patients and upper or lower scalp (especially neonates) for generally pediatric patients [17].

**Data processing and analysis:** data were entered into the computer using Epi Data version 3.1 and transported to Statistical Package for Social Sciences (SPSS) version 25.0 for analysis. Descriptive and inferential statistics were analyzed and presented. Initially, bivariate logistic regression was carried out to explore the association of each independent variable with the outcome variable. Thereafter, to see the relationship between practice and socio-demographic and other variables, multivariable logistic regression was used. Variables with a P-value ≤0.2 in the bivariate logistic regression were used in the multiple logistic regression analysis. P-value ≤0.05 and 95% confidence level were considered statistically significant.

**Ethics approval and consent to participate:** an ethical clearance letter was obtained from the Research Ethics Committee of Debre Tabor University College of Health Sciences (Reference number: 972/2014 E.C./CHS). The permission and agreement consent were obtained from the study hospitals before the study after an explanation of the purpose of the study through a support letter. This study was conducted per the Declaration of Helsinki. Each study participant was well informed about the aim of the study, benefits, and risks. Informed written consent was secured from study participants; study participants´ confidentiality was maintained; no personal identifiers were used in the data collection questionnaire, and codes were used in place of them; the recorded data were not accessed by a third person, except the researcher; and data sharing will be enacted based on the consent and permission of research participants.

## Results

**Socio-demographic characteristics of the respondents:** three hundred and ninety respondents completed the questionnaire, with a response rate of 93.98%. The study found 207 (53.1%) were male and the rest were female. Most, 315 (80.8%) of the respondents belong to the age group of 21-34 years. The majority, 350 (89.7%) of the respondents were Bachelor's degree holders and 135 (34.6%) respondents had work experience of less than 5 years and 64 (16.4%) of them had more than 10 years of work experience (Table 1).

**Table 1:** socio-demographic characteristics of nurses and midwives working in Referral Hospitals of Amhara Region, Ethiopia, 2022

Variables		Frequency	Percentage
**Sex of respondents**	Male	207	53.1
	Female	183	47.9
**Age of respondents**	21-34	315	80.8
	35-49	70	17.9
	50-65	5	1.3
**Level of education**	Diploma	39	10
	Bachelor of degree	351	90
**Work experience**	<5 years	135	34.6
	5-10 years	191	49
	>10 years	64	16.4

**Practice of nurses and midwives on the care of peripheral intravenous cannulation:** in this study, only 4.9% of participants had good practice but, 95.1% of the respondents had poor practice on the care of peripheral intravenous cannulation. Nurses´ and Midwives practice on the care of peripheral intravenous cannulation working in Referral Hospitals of Amhara Region, Ethiopia, 2022 (Table 2). In the practice part of this study, observation had conducted on 41 participants as they insert PIVC to discover the natural practice with the procedure. More than half of the observations did not wash their hands or scrub their hands using alcohol before the procedure of intravenous cannulation; a small number of observations explain the procedure to their client before insertion. Almost none of the observations open the IV kit before the procedure. In this observation, the frequently used vein site was the left forearm (21.9%) and the failure rate was 9.6% (Table 3).

**Table 2:** practice of IV cannula care and maintenance

Variables	Yes, n (%)	No, n (%)
Do you always maintain an aseptic technique during preparing, inserting, and removing of IV cannula?	309(79.2)	81(20.8)
Do you always use transparent dressing when securing an IV cannula?	39(10)	351(90)
Do you always write the date, time, site, size, due date change, and name of the person cannulated?	178(45.6)	212(54.4)
Do you always educate your patient on how to care for the IV cannula?	304(77.9)	86(22.1)
Do you always educate Your patient on how to recognize the signs and symptoms of IV cannulation infection?	243 (62.1)	147(27.9)
When placing a peripheral Cannulation, if you are unable to access the vein after two attempts, as the next step should you find the nurse or midwife with the best skills to evaluate the patient’s veins?	282(72.3)	108(27.7)
Do you perform a daily assessment, cleaning of the site, and check the patency of the IV cannula after insertion?	247(63.3)	143(36.7)
When you see there is a sign of complication like redness of the site, do you immediately change the IV cannula to a non-affected part?	339(86.9)	51(13.1)
Do you always change the dressing when it is wet or dislodged?	312(80)	78(20)
Do you always change the IV cannula after 12-72 hours of insertion?	162(41.5)	228(58.5)
Do you use the administration set (iv set) within 72 hours without disconnecting and immediately throw it if detached from the cannula?	165(42.3)	225(57.7)
Is there any guideline/protocol for peripheral intravenous cannulation in your institution? If no skip	16(4.1)	374(95.9)
Have you ever trained for the care and maintenance of peripherally inserted intravenous cannulation?	14(3.6)	376(96.4)

**Table 3:** nurses and midwives practice during observation working in Referral Hospitals of Amhara Region, Ethiopia, 2022 (n=41)

Procedures/variables	Performed correctly, n (%)	Not performed correctly, n (%)
Verify physicians order	28(68.3)	13(37.3)
Assemble and organize equipment		
Alcohol	26(63.4)	15(36.6)
Gloves	41(100)	0(0)
Tourniquet	4(9.8)	37(90.2)
Suitable Iv cannula	41(100)	0(0)
Plaster	41(100)	0(0)
Syringe	20(48.8)	21(51.2)
Saline	18(43.9)	23(56.1)
Gauze	27(65.9)	14(34.1)
Small towel	2(4.9)	39(95.1)
Wash hands/scrub hands using alcohol	15(36.6)	26(63.4)
Explained the procedure to the patient	9(21.9)	32(78.1)
Select the appropriate catheter and open the IV kit	2(4.9)	39(95.1)
Prepare saline flash for IV lock	21(51.2)	20(48.8)
Vigorously use circular motion at the site from the center outward for at least 30 seconds using 70% alcohol	7(17.1)	34(82.9)
Allow the agent to completely air-dry	9(21.9)	32(78.1)
When the catheter is in place, carefully place one 2_2 gauze under the catheter and needle	2(4.9)	39(95.1)
Release tourniquet	41(100)	0(0)
Flush catheter with 0.9% saline	23(56.1)	18(33.9)
Clean the site of moisture and blood	23((56.1)	18(33.9)
Place film over the insertion site and up to the catheter hub. Pinch the film (plaster) around the catheter hub to secure it.	41(100)	(0)
Label tubing according to hospital policy. Label all fluids with the date, time hung, medication additives, and initials.	9(21.9)	32(78.1)
Dispose of all the equipment	41(100)	0(0)
Ensure the patient is comfortable and thank them	23(56.1)	18(43.9)
Hand washing/hand scrubbing using alcohol	11(26.8)	30(73.1)
Document in nurse’s and midwives’ notes procedure and patient response	25(61)	16(39)

**Factors associated with nurses´ and midwives´ care on peripheral intravenous catheter:** in this study, nurses and midwives who have greater work experience were seven times (adjusted odds ratio [AOR]= 7.269, 95%CI= (1.68-31.40) more likely to have good practice on the care and maintenance of PIVC and nurses and midwives who had special training on care of PIVC have 10 times AOR= 10.12, 95%CI= (4.32-19.45) more likely to have good practice on the care and maintenance of PIVC (Table 4).

**Table 4:** logistic regression analysis for the practice of nurses and midwives on the care of peripheral intravenous cannulation in Referral Hospitals of Amhara Region, Ethiopia, 2022

Variables		Care of PIVC		COR 95%CI	AOR (95%CI)
		Poor, n (%)	Good, n (%)		
**Sex**	Female	155(39.7)	28(7.2)	0.65(.329,2.922)	0.523(.310,5.1)
	Male	156(40)	51(13)	1	1
**Level of education**	Bachelor	276(70.8)	75(19.2)	2.378(.819,6.901)	3.024(0.8,9.07
	Diploma	35(9)	4(1)	1	1
**Work experience**	>10 years	57(14.6)	7(1.8)	4.02(1.13, 14.28)	7.26(1.68, 31.40)*
	5-10 years	183(46.9)	8(2)	1.43(.42, 4.85)	1.54(.45, 5.23)
	<5 years	131(33.6)	4(1%)	1	1
**Presence of guideline**	No	368(94.4)	6(1.5)	1	1
	Yes	3(.8)	13(3.3)	6.78(0.9, 11.81)	3.31(2.83, 12.87)*
**Trained in the care of PIVC**	No	367(94.1)	9(2.3)	1	1
	Yes	4(1)	10(2.6)	11.1(3.3,20.3)	10.1 (4.32.19.45)*

Note: *stands for p value ≤0.05

## Discussion

This study finding showed that 4.9 at 95% CI (3,7) of respondents had good experience taking care of intravenous cannulations that were inserted peripherally. This study's findings were less than those of research carried out in Bangladesh and Nepal [14,18]. Approximately 43% of nurses and midwives were aware of the proper vein to insert; this is less than the Malaysian study's findings and does not align with the American CDC's 2011 advice [15,17]. The aseptic non-touch technique, which avoids touching vital equipment and body parts, is a crucial infection prevention strategy in resource-constrained environments like ours. However, nearly half of the study's responders (44.6%) were unaware of important details that are not touched during infusion therapy [19]. This study revealed, among other things, that most participants (56.6%) were aware of the possibility of having PIVC placed in adult patients' or clients' lower limb veins; these findings are validated by other research [17,20]. Around 41.5% of participants in this study were replacing their IV cannulas after 12-72 hours, despite the fact that several studies have revealed conflicting findings about the optimal timing for PIVC removal and replacement that is, whether replacing an IV cannula on a regular basis or only when clinically necessary to avoid difficulties. However, the majority of study participants (90%) did not utilize transparent dressings. In this scenario, it can be challenging to grade and assess the clinical status of the IV site, making it challenging to follow clinically recommended replacement and removal. Thus, it's crucial to replace IV cannulas on a regular basis [21].

The goal of the study was to observe 41 nurses and midwives in order to corroborate the answers provided in the practice questionnaire. The outcome revealed inadequate practice in every area. There had been partial maintenance of the aseptic procedure. Approximately 63.3% of participants complete the questionnaire while using the cannula to preserve patency every day; nevertheless, during observation, 56% of participants flash the cannula by 0.9% NS.

In this study, some independent variables are associated with the practice of nurses and midwives in the care of PIVC. Work experience, the presence of guidelines, and training significantly affect the practice of nurses and midwives. The study conducted in Malaysia showed that the respondents who possessed a bachelor deemed to score good practice [22]. Similarly, a study conducted in Nepal showed that socio-demographic data like level of education and work experience influence the practice level of the respondent [14]. In this study, training influenced the practice of respondents, which was similar to previous studies conducted in Egypt, America, and London [14,23,24]. The presence of guidelines in the institution has also influenced good practice in the care of PIVC which is in line with previous studies in Malta and China [23,24].

## Conclusion

Work experience, presence of guidelines and training of the care of PIVC were positive predictors for good practice in the care of PICV. Based on the observational result of this study, an aseptic technique was not maintained and full access to equipment for the insertion of PIVC was not available which magnifies nurses and midwives applying poor practice in the care of PIVC. Unless nurses and midwives perform good practice in PIVC, they will introduce microorganisms and may end up with complications related to the procedure which endangers patients´ health and increases morbidity, mortality, and hospital stay. Therefore, nurses and midwives shall update themselves on the care of peripheral intravenous cannulation, and also hospitals should provide quality and targeted training, comprehensive guidelines, and fulfill equipment for peripheral intravenous cannulation.

### What is known about this topic

- *Proper care and maintenance of peripheral intravenous cannulas are critical in preventing phlebitis, infection and thrombosis which can be significantly reduced by best practice of adherence;*
- *Although nurses and midwives are trained in intravenous cannulation care and maintenance, there may be inconsistencies in the application of best practices due to variations in training programs and continued education;*
- *Even though numerous health organizations have published guidelines that outline appropriate techniques for IV insertion, maintenance and care, gaps in practice often persist*.

### What this study adds

- *This is the first study in the study area to estimate the prevalence of practice towards care and maintenance of peripheral intravenous cannula among nurses and midwives in teaching hospitals and its predictors;*
- *By identifying the prevalence and specific intravenous cannulation care and maintenance-related factors among nurses and midwives in this region, the study highlights pressing areas for improvement and targeted training interventions;*
- *This study lays the groundwork for future research into interventions aimed at enhancing the competence and confidence of nurses and midwives in performing IV cannula care and maintenance*.
